# Understanding non-compliance to colorectal cancer screening: a case control study, nested in a randomised trial [ISRCTN83029072]

**DOI:** 10.1186/1471-2458-5-139

**Published:** 2005-12-22

**Authors:** Paolo Giorgi Rossi, Antonio Federici, Francesco Bartolozzi, Sara Farchi, Piero Borgia, Gabriella Guasticchi

**Affiliations:** 1 Agency for Public Health, Lazio Region. Via di S. Costanza, 53 Rome, 00198, Italy; 2 Campus Biomedico, University Hospital. Rome, Italy

## Abstract

**Background:**

The major limit to colorectal cancer screening effectiveness is often low compliance. We studied the reasons for non compliance and determinants of compliance to faecal occult blood tests in Lazio, Italy.

**Methods:**

This is a case-control study nested within a trial that tested the effect of type of test and provider on colorectal cancer screening compliance. Non compliant trial subjects were classified as cases, and compliant subjects were classified as controls. We sampled 600 cases and 600 controls matched by their general practitioner, half were invited for screening at the hospital, and the other half directly at their general practitioner's office. Cases and controls answered questions on: distance from test provider, logistical problems, perception of colorectal cancer risk, confidence in screening efficacy, fear of results, presence of colorectal cancer in the family, and gastrointestinal symptoms.

**Results:**

About 31% of cases never received the letter offering free screening, and 17% of the sampled population had already been screened. The first reported reason for non-compliance was "lack of time" (30%); the major determinant of compliance was the distance from the test provider: odds ratio >30 minutes vs <15 minutes 0.3 (95%CI = 0.2–0.7). The odds ratio for lack of time was 0.16 (95% IC 0.1–0.26). The effect was stronger if the hospital (0.03 95%CI = 0.01–0.1) rather than the general practitioner (0.3 95%CI = 0.2–0.6) was the provider. Twenty-two percent of controls were accompanied by someone to the test.

**Conclusion:**

To increase compliance, screening programmes must involve test providers who are geographically close to the target population.

## Background

The efficacy of colorectal cancer screening (CRCS) using faecal occult blood tests (FOBT) in reducing colorectal cancer (CRC) mortality in the 50–75 year old population has been demonstrated in large randomised trials[1]. The reduction in mortality by screening is strictly linked to its ability to involve as many people among the target population as possible. The scientific literature about the reasons for non compliance has generated few definitive operational recommendations [2-6]. Some reviews have summarized the knowledge and indications that have emerged from the literature[4,5,7]. Some studies have identified several factors associated with higher prevalence of FOBT use: presence of symptoms and a family history of CRC are associated with higher prevalence, while the opposite is true for smoking habits. No consistent relation with gender or socio-economic status has been observed. Other indications come from intervention studies, and the recommendations are consistent: the involvement of one's own physician, phone and mail reminders, and one sample tests all increase compliance. Some prospective studies have found that a previous FOBT was a predictor of compliance, while some studies have found that participation decreased after 75 years of age. Most studies tested a hypothesis based on the Health Belief Model[8]. Finally, several surveys have studied the reasons for non compliance: practical reasons ranked first in most of the studies; not having health problems was also a frequent answer, along with anxiety and embarrassment.

The Agency for Public Health of Lazio, Italy, designed a series of studies in order to implement an evidence-based CRCS program; a special focus was given to the barriers to screening, and how to obtain high screening compliance [9-12].

In this study we analysed self-reported reasons for non compliance, individual and environmental determinants of screening compliance, and the interaction between them.

## Population and methods

### Setting

Lazio has 5.3 million inhabitants and includes the metropolitan area of Rome (2.9 million). The CRCS target population (50–74 year olds) is 1.5 million. The health service is organised into 12 Local Health Units, which include 50 districts.

### The study design (figure 1)

**Figure 1:**
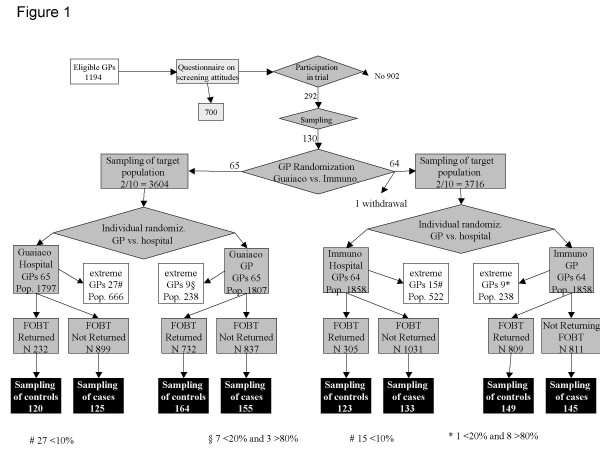
**Study designs**. The figure illustrates the design of the three linked studies. The white box represents the reference population; the light grey boxes refer to the survey; the dark grey boxes to the trial; and the shadowed boxes to the case control study.

The study design has been described elsewhere[9]; here we summarize the principal characteristics. We selected an opportunity sample of 13 hospitals, out of 20 who will participate in the screening programme in the near future, in order to represent all types of gastroenterology units (5 university hospitals, 2 large research hospitals, 6 local hospitals) and all geographic areas (7 in the metropolitan area of Rome, 2 in the outskirts of Rome, 4 in towns and small cities of the province). From June 2002 to April 2003, all GPs with an office in the 13 hospital districts selected (1192) were surveyed[10] and asked to participate in the randomised trial.

In each of the 13 districts, we sampled 10 GPs of the 24.5% (292/1194) who had agreed to participate. The sampled GPs were randomised as follows: in each district, five were assigned to the immunochemical test and five to the Guaiac test (Guaiac Hemo-Fec, Roche Diagnostic, Mannheim Germany, and immunochemical OC-Hemodia, Eiken, Tokio Japan, distributed by Alpha Wasserman, Milan Italy). We sampled 2/10 of the target practice population for each GP; 1/10 of the GP's beneficiaries were randomised to the GP arm and 1/10 to the hospital arm. The coordinating centre mailed a letter to the population sample: for the half randomised to the GP arm, the letter invited patients to pick up and return the FOBT at the GP's office; for the half randomised to the hospital arm, the letter invited the patient to pick up and return the FOBT at the hospital. The study was submitted and approved by the Committee for Ethics in Screening of the Regional Agency for Public Health, 16th June 2002, approval n° 1. Informed consent is not required for this type of study.

**Figure 2:**
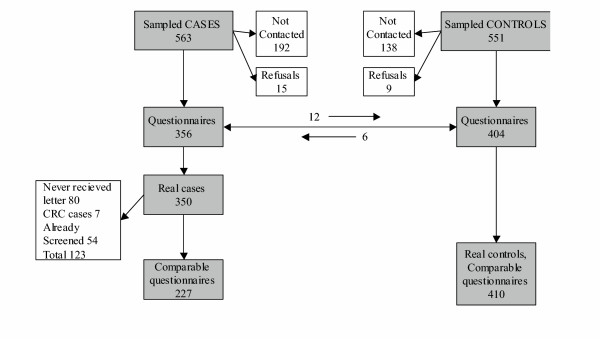
**Questionnaire response rate**. Response to the questionnaire and technical obstacles to screening compliance. The arrows show 12 cases reclassified as controls and 6 controls reclassified as cases during the interview.

We defined the non-compliant population as cases, and the compliant population as controls. We sampled about 600 cases and 600 controls matched by GP and arm of randomisation. The matched design was imposed to exclude practices with too high or too low compliance because in these extreme situations we had a strong imbalance between cases and controls. We excluded practice populations with compliance lower than 20% and higher than 80% in the GP arm and practice populations with a compliance lower than 10% in the hospital arm (figure 2).

For the 1200 people sampled we looked for updated telephone numbers and delivered brief telephone questionnaires about the reasons for non-compliance to the cases, and about the reasons for compliance to the controls. We inquired about distance from the provider, logistical problems, perception of CRC risk, confidence in screening efficacy, fear of the results, family history of CRC, and gastrointestinal symptoms[13].

### Analysis

Demographic factors, gender, age and residence, were analysed using the entire population participating in the trial, i.e. 7309. All the other information was available only for the sample interviewed.

When analysing the trial population, logistic models were constructed taking into account the effect of the GP clusters; when analysing the case control sample, we adjusted for GP and provider. We tested the goodness of fit of the models using the Hosmer and Lemeshow test for deciles of probability[14]. All analyses were performed using Stata 7.0 statistical software[15]. Residence was categorised as follows: Rome, cities with a gastroenterology centre, and cities without a centre. We also categorized the cities based on the number of inhabitants. We alternated the two in a logistical model and compared the pseudo r squared and the goodness of fit.

### Sample size and power of the studies

#### Trial

The study size was calculated for the main objective of the trial and is described elsewhere[9]. The study has a power of 95%, with alpha 0.05, to detect a 20% difference in compliance between the less represented age class and the rest of the sample. The power reduction due to provider interaction, and to the subsequent stratified analysis, allowed us to detect a difference of 27%.

#### Case control

The sample size was calculated to obtain a power of 80%, with alpha .05, to detect a difference in exposure prevalence of 1/3 and a lowest prevalence of exposure of 30%. The expected response rate was 60%. The resulting sample size was 600 cases and 600 controls.

## Results

### The never-reached

**Figure 3:**
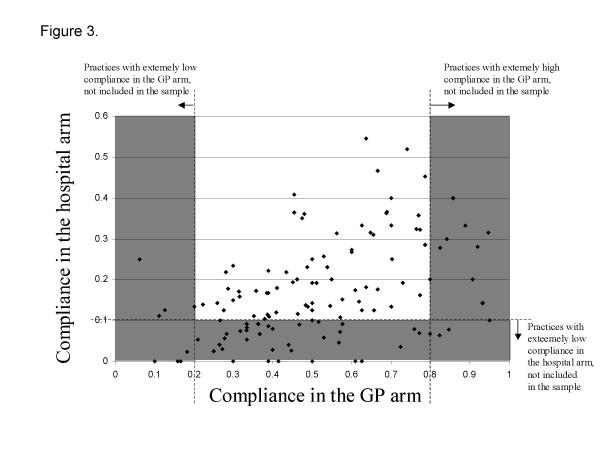
**General Practitioners' exclusion criteria**. General Practices which obtained extremely low or extremely high compliance during the trial (grey areas), were excluded from the survey on reasons for compliance or non compliance.

Figure 2 shows the compliance of cases and controls to the case-control interview. The percentage of people not reachable for the interview was higher among the non compliant than in the compliant group (34.1% vs. 25.0%). The percentage of refusals shows the same group differences but is lower in both groups (2.7% vs. 1.6%). Eighty of the interviewed people who were non-compliant to screening (22.5%) declared not having received any letter inviting them to participate in the programme and they were excluded from the following analysis (figure 3).

### The already-reached

Fifty-four non compliant people declared that they had already been screened, 20 by FOBT in the previous two years, 38 by colonoscopy in the previous 10 years and 21 by double contrast barium enema (DCBE) in the previous 10 years (some people had more than one test). They were excluded from the following analysis. On the other hand, we found 82 controls who had already been screened: 11 by FOBT, 60 by colonoscopy, and 28 by DCBE. These controls were included in the analysis. None of the interviewed people declared to have had a sigmoidoscopy. The 136 people who had already been screened, when compared with the entire interviewed population, were slightly more likely to be male (55.2%; 95%CI 46.4–63.8), to live outside of Rome (46.7%; 95%CI 38.3–55.8), were significantly older (test for linear trend chi2(1) = 6.93; Pr>chi2 = 0.0085), had a significantly higher prevalence of gastrointestinal symptoms (39.0%; 95%CI 30.7–47.7), more CRC cases among relatives (16,2%; 95%CI 10.4–23.4), and were more likely, with border-line significance, to agree to a new FOBT screening (OR 1.5; 95%CI 1.0–2.3; adjusted for GP, provider, age, gender and residence).

### Analysis of demographics

We used the entire population involved in the trial, 7320 individuals, to analyse the effect of most demographic factors. Women were significantly more compliant, although the size of this effect is small. Compliance increased in men with age until 65–69, and than decreased slightly; among women we did not observe any clear age trend. There was higher compliance outside Rome in medium-sized towns with gastroenterology centres, while people living in small towns and rural areas without centres showed low compliance. Table 1 shows the figures described above and reports the provider effect, i.e. hospital and GPs, tested in the trial.

**Table 1:** Logistic regression for determinants of non compliance, data from randomised controlled trial. All the Odds Ratios are adjusted by age, residence, provider and age if possible.

	total				males				females			
			
	FOBT screening		OR	95%CI	FOBT screening		OR	95%CI	FOBT screening		OR	95%CI
									
	no	yes			no	yes			no	yes		
**demographics**												
Gender*												
male	2269	1099	1	-								
female	2585	1342	1.12	1.0–1.3								
												
Age*												
50–54	1126	464	1.00	-	539	201	1.0	-	581	262	1.0	
55–59	916	457	1.34	1.2–1.6	457	202	1.4	1.1–1.9	456	255	1.3	1.0–1.6
60–64	950	449	1.27	1.1–1.5	440	194	1.4	1.1–1.9	510	255	1.1	0.89–1.4
65–69	844	460	1.44	1.2–1.7	386	229	1.8	1.4–2.3	457	231	1.2	0.94–1.5
70–74	738	326	1.22	1.0–1.5	317	140	1.5	1.1–2.0	421	186	1.1	0.82–1.4
												
**logistics and demographics**												
Residence*												
Rome	2832	1285	1.00	-	1284	573	1.0	-	1540	710	1.0	
towns with gastroenterology unit	1327	763	1.55	1.1–2.2	652	322	1.6	1.2–2.2	673	441	1.7	1.3–2.2
other towns	566	333	1.13	0.73–1.7	265	174	1.2	0.73–1.9	299	159	1.1	0.70–1.8
												
**logistics**												
provider												
hospital	3028	600	1.0		1388	276	1.0		1631	324	1.0	
GP	1838	1843	6.0	5.3–6.8	881	823	5.7	4.7–6.8	954	1018	6.3	5.3–7.4

* For 14 people gender was unknown, for 579 age was unknown, for 203 residence was unknown.

We also considered employment status, information only available for the case control study sample. Employed and retired people had the same low level of compliance, while homemakers had a significantly higher level of compliance (OR 2.2; 95%CI 1.3–3.7; table 2).

**Table 2:** Logistic regression for determinants of non compliance, data from questionnaire. All the Odds Ratios are adjusted by age, gender and provider.

	non compliant	compliant	OR*	95% CI	
**demographics**					
employment status					
currently employed	69	91	1		
homemaker	76	223	2.2	1.3	3.7
retired	53	81	1.1	0.6	1.9
unemployed	2	5	1.8	0.3	9.6
m.i.	27	10			
					
**psychological and cultural**					
educational level					
0–4 years of study	18	17	1		
5–7 years of study	53	119	2.5	1.2	5.2
8–12 years of study	35	83	2.5	1.1	5.6
high school graduate	49	117	2.7	1.3	6.0
university	24	46	2.1	0.9	5.1
m.i.	48	28			
					
Gastrointestinal symptoms					
no	198	291	1		
yes	29	119	2.7	1.7	4.2
					
CRC cases among relatives					
No	212	351	1		
yes	15	51	1.8	1.0	3.2
					
**logistics**					
lack of time	69	25	0.2	0.1	0.3
					
distance					
1–15 min	122	301	1		
15–30	44	75	0.8	0.5	1.3
>30	21	15	0.3	0.2	0.7
m.i	40	19			
					
type of transportation					
public	31	29	1		
private	117	218	2.1	1.2	3.7
on foot	67	159	2.1	1.1	4.1
m.i.	12	4			
					
**Analysis stratified by provider:**					
in the **hospital **arm					
lack of time	32	4	0.04	0.01	0.12
					
in the **GP **arm					
lack of time	37	21	0.3	0.2	0.6

### Reasons for non-compliance

Only 24 people of the 350 interviewed (6.9%) picked up the test but did not return it, consequently we did not perform separate analyses for initial agreement and actual compliance. Very few people answered the proposed justifications for non-compliance. Table 3 shows the justifications given by the non-compliant, multiple choice answers selected are indicated with a star, and others were summarized from the open answers. The most frequent answer was "lack of time" (30%), followed by "feel healthy" (8%) and "ill during the study" (6%). Less than ten people chose any of the other answers. About 40% did not respond. Nine people declared to be covered but did not report how or when they were last tested.

**Table 3:** Reason for non compliance

reasons for non compliance to the screening	N	%
total	227	
none declared	89	39.2
*lack of time	70	30.8
*feel healthy	18	7.9
ill	14	6.2
*it was impossible to contact the provider	9	4.0
already covered	9	4.0
do not want	7	3.1
*anxiety over outcome	6	2.6
*the letter was not clear about what I was expected to do	6	2.6
*embarassed by test execution	6	2.6
away from home during study	4	1.8
*fear about risks of the test	3	1.3
oversight	3	1.3
*negatively advised	1	0.4
bleeding (hemorroids)	1	0.4

* Proposed aswers

### Logistical barriers

We observed compliance that was three times higher at GP's office than at hospital gastroenterology centres. There was one gastroenterology centre per district, while there were 10 GPs' offices per district. In the case control study we compared the travelling time to the GP office and to the hospital in the two study arms: 84% stated it took less than 15 minutes to reach the GP, 12% reported 15–30 minutes, and 4% >30 minutes, while in the hospital arm, 58% stated less than 15 minutes, 29% 15–30 minutes, and 13% >30 minutes (test for linear trend chi2(df1) = 55.3; P < 0.00005). Also in the multivariate analysis, the time required to reach the test provider was a strong determinant of compliance: odds ratio of 15–30 minutes versus <15 minutes 0.8 (95%CI 0.5–1.3) >30 minutes versus <15 minutes 0.3 (95%CI 0.2–0.7). We compared also the odds of people declaring "lack of time" to perform the test in the compliant and non compliant populations: the odds ratio was 0.16 (95%CI 0.1–0.26) indicating that it is strongly associated with non compliance. The provider was an effect modifier of the "lack of time" barrier: the odds ratio is 0.03 (95%CI 0.01–0.1) in the hospital arm and 0.3 in the GP arm (95%CI 0.2–0.6) (table 2).

### Cultural, psychological and emotional barriers

The effect of the educational level was observed for people with fewer than 5 years of education. Anxiety regarding results was reported by 17.8% of the compliant population, fear of the test by 8.8%, and embarrassment by 3.9%. Having gastrointestinal symptoms at the moment of contact is a determinant of compliance: odds ratio 2.7 (95%CI 1.7–4.2). An effect with borderline statistical significance was also observed for people with a family history of CRC: odds ratio 1.8 (95%CI 1.0–3.2).

### Chaperones

Ninety (22%) of the 410 compliant people interviewed were accompanied by someone to the hospital or GP office: 86 by a relative, 2 by a friend, and 2 by an attendant. There was a slightly higher proportion of people >70 years old (31%), without a clear age trend, and of females (25%), although neither was statistically significant. Chaperones were more common outside of Rome (27%, chi2 = 4.6, p = 0.033) and when the hospital was the provider (34%, chi2 24.8, p < 0.0005).

## Discussion

### Limits

The response rate for the case control is acceptable, while the participation rate among the GPs is very low and generates several concerns regarding the validity of the overall observed compliance. The difficulty in involving GPs is a well-known problem and can reduce study validity[16]. Nevertheless the comparisons between compliant and non-compliant populations are not affected by the GPs' self-selection; these comparisons are more likely to be biased on the differences in response rates between cases (non-compliant to FOBT) and controls (compliant to FOBT).

We did not design this study to be a survey of the Lazio population, although the population sampled for the trial and for the nested case control was a large sample, which was not self-selected.

In designing the study we overestimated the power of the case control. In fact, of 356 non-compliant case interviews, only 227 were informative.

Because of the low percentage in the trial of people that picked up the test but did not return it (8.6%), and consequently also in the case control sample (6.9%), we did not perform a separate analysis on initial agreement to participate in screening and actual compliance. We can only affirm that the immunochemical test led to a higher percentage of returned tests than the guaiac test[11].

The questionnaire may underestimate cultural barriers like fear of the test or anxiety about the results, both due to lack of awareness of the people interviewed, and to the limits of telephone interviews [4].

### Never-reached

A high proportion of not reached people means the screening programme was inequitable, and due to administrative mistakes denied some their opportunity to be tested. This group can be estimated as the people who did not receive the letter, plus the difference between not contacted cases and not contacted controls. We estimated that 22% + (34% – 25%) = 31%; about one-third of the non compliant population (about 20% of target population) was not reachable by letter. We had only self-reported information about receiving the letter, and could not verify if in fact the letter had been delivered to the right address and then thrown away. From mammography screening experience, we know that anywhere from 3% to 18% of letters were returned to sender. This was the most relevant barrier we observed for the screening programme, an issue which other researchers have also highlighted [17-19].

### Subjects reached

This case control study, with the limits described above, is the first survey in our region to explore CRC screening coverage. The proportion of people screened with one of the recommended tests is 15.4% among non compliant and 19.8% among compliant people, and considering that 35% of the general population is compliant, we estimate 17% coverage with an unknown confidence interval. This coverage level is lower than what was found in the USA[20]. The absence of flexosigmoidoscopy in our sample reflects the scarce use of this technique by Italian endoscopists, although some may confuse sigmoidoscopy with colonoscopy. Colonoscopy is the most common test, accounting for 70% of coverage, and consequentially may explain the very low level of coverage. The screening of high risk groups, such as people with symptoms or with family history of CRC, for whom colonoscopy (or DBCE) is recommended by the guide-lines[21], represents about one half of the covered population (47.8%).

### Reported motivations for non-compliance

Many people did not provide a motive for non-compliance to screening, and those who did often did not use the answers provided. This may be one of the limits of the questionnaire used: Closed questions with a long list of multiple choices are not well adapted with telephone interviews. Furthermore the proposed answers were based on the Health Belief Model[8], which does not adapt well to the realities screening non-compliance in our setting. The most common justification was "lack of time" followed by feeling healthy, two indicators of a low perception of susceptibility, rather than of the severity or of the perception of risks and benefits of the test[22,23].

### Logistical barriers

Our results show that the major determinant of non compliance to CRC screening was the "lack of time". We interpret all of the following factors as time-related: 1) the higher compliance found among women and homemakers in particular, consistent with other Italian studies[13]; 2) the higher compliance found in medium-sized towns with a gastroenterology unit which are the easiest places to be tested; 3) higher compliance found among subjects allocated to the GP arm, where the doctor's office is more easily reachable than the gastroenterology centre, where "reachable" may be a mix of familiarity, distance, and expected waiting time; 4) the single evacuation test (immunochemical) obtained a 20% higher compliance than the three evacuation test (Guaiac) [11]; 5) more time was reportedly required to reach the provider in the non compliant group than in the compliant group; 6) finally, the lowest odds ratio was observed for "lack of time", and the most frequent justification was "lack of time".

Subjects who reported to live close to the provider were more likely to comply. While in the majority of studies that have addressed this topic[5] the logistical reasons for non compliance have ranked first, the effect of distance from the test provider has not yielded consistent results[24,25]. In our study the self-reported time to reach the test provider reflects perceived distance that may reflect the degree of willingness to perform the test; from this point of view it is not surprising that the effect of perceived distance on compliance was more important in the hospital arm than in the GP arm. This finding suggests that interventions should make it easier for patients to understand how to incorporate this important and non time-consuming task into their lifestyle.

### Cultural, psychological and emotional barriers

In our study we observed the paradox that fear of the test and anxiety over the results were more frequent in those who took the test than in those who did not. The problem could be with the timing of the interview. We contacted the compliant group after testing, so the questions were connected to something that had already occurred; the non compliant population had to base their answers on a hypothetical situation[4,22].

Other reasons for our difficulties in understanding the psychological and cultural barriers may have been the poor fit of the Health Belief Model we used. Other authors have found similar difficulties using this model[22,26].

Nevertheless there are indications that cultural and psychological barriers exist: 1) the underestimation of the problem is evident in most of the reasons given by the non compliant, and confirmed by the strong positive effect the presence of symptoms and family history of CRC have on compliance; 2) the anxiety produced by screening, as well as logistical problems of being screened, may be inferred from the high percentage of people who were accompanied to screening; this group surprisingly consisted not only of old women, but also middle-aged men.

## Conclusion

To increase compliance, screening programmes must make all efforts possible to involve test providers who are geographically close to the target population.

Our population suggested one way to overcome logistical and psychological barriers may be to invite all target individuals from a single household or block for testing on the same day[27].

## Competing interests

The author(s) declare that they have  no competing interests.

## Authors' contributions

PGR was involved in conceiving and designing the study, the statistical analysis and writing the paper. FB was involved in designing and conducting the study. AF was involved in conceiving and designing the study and writing the paper. SF was involved in designing the study and statistical analyses. PB was involved in conceiving and designing the study and writing the paper. GG was involved in conceiving the study and writing the paper. All authors read and approved the final manuscript.

## Pre-publication history

The pre-publication history for this paper can be accessed here:



## References

[B1] Towler B, Irwig L, Glasziou P, Kewenter J, Weller D, Silagy C (1998). A systematic review of the effects of screening for colorectal cancer using the faecal occult blood test, Hemoccult. BMJ.

[B2] Box V, Nichols S, Lallemand RC, Pearson P, Vakil PA (1984). Haemoccult compliance rates and reasons for non-compliance. Public Health.

[B3] Farrands PA, Hardcastle JD, Chamberlain J, Moss S (1984). Factors affecting compliance with screening for colorectal cancer. Community Med.

[B4] Blalock SJ, DeVellis BM, Sandler RS (1987). Participation in fecal occult blood screening: a critical review. Prev Med.

[B5] Vernon SW (1997). Participation in Colorectal Cancer Screening: a Review. J Nat Cancer Inst.

[B6] Jepson R, Clegg A, Forbes C, Lewis R, Sowden A, Kleijnen J (2000). The determinants of screening uptake and interventions for increasing uptake: a systematic review. Health Technol Assess.

[B7] Schoen RE (2002). The case for population-based screening for colorectal cancer. Nat Rev Cancer.

[B8] Rosenstock IM (1975). Patients' compliance with health regimens. JAMA.

[B9] Giorgi Rossi P, Federici A, Bartolozzi F, Farchi S, Borgia P, Guastcchi G (2005). Trying to improve the compliance to colorectal cancer screening: a complex study design for a complex planning question. Cont Clin Trial.

[B10] Federici A, Giorgi Rossi P, Bartolozzi F, Farchi S, Borgia P, Guastcchi G (2005). Survey on colorectal cancer screening knowledge, attitudes and practices of general practice physicians in Lazio, Italy. Prev Med.

[B11] Federici A, Giorgi Rossi P, Borgia P, Bartolozzi F, Farchi S, Gausticchi G (2005). The immunochemical faecal occult blood test leads to higher compliance than the guaiac for colorectal cancer screening programs: a cluster randomised controlled trial. J Med Screen.

[B12] Federici A, Giorgi Rossi P, Bartolozzi F, Farchi S, Borgia P, Guasticchi G (2006). The role of GPs in increasing compliance to colorectal cancer screening: a randomised controlled trial. Cancer Causes and Control.

[B13] Segnan N, Senore C, Andreoni B, Aste H, Bonelli L, Crosta C, Ferraris R, Gasperoni S, Penna A, Risio M, Rossini FP, Sciallero S, Zappa M, Atkin WS (2002). SCORE Working Group – Italy. Baseline findings of the Italian multicenter randomized controlled trial of "once-only sigmoidoscopy" – SCORE. J Natl Cancer Inst.

[B14] Hosmer DW, Lemeshow S (2001). Applied Logistic Regression.

[B15] Stata Corporation (2001). Stata statistical software: release 7.0.

[B16] Schoenman JA, Berk ML, Feldmann JJ, Singer A (2003). Impact of differential response rate on the quality of data collected in the CTS physician survey. Eval Health Prof.

[B17] King J, Fairbrother G, Thompson C, Morris DL (1992). Colorectal cancer screening: optimal compliance with postal faecal occult blood test. Aust N Z J Surg.

[B18] Ornstein SM, Musham C, Reid A, Jenkins RG, Zemp LD, Garr DR (1993). Barriers to adherence to preventive services reminder letters: the patient's perspective. J Fam Pract.

[B19] Brunton M, Thomas DR (2002). Privacy or life: how do women find out about screening mammography services?. N Z Med J.

[B20] Seeff LC, Nadel MR, Klabunde CN, Thompson T, Shapiro JA, Vernon SW, Coates RJ (2004). Patterns and predictors of colorectal cancer test use in theadult U.S. population. Cancer.

[B21] American Gastroenterological Association (1997). Colorectal cancer screening: clinical guidelines and rationale. Gastroenterology.

[B22] Macrae FA, Hill DJ, St John DJ, Ambikapathy A, Garner JF (1984). Predicting colon cancer screening behavior from health beliefs. Prev Med.

[B23] Spector MH, Applegate WB, Olmstead SJ, DiVasto PV, Skipper B (1981). Assessment of attitudes toward mass screening for colorectal cancer and polyps. Prev Med.

[B24] Bulliard JL, de Landtsheer JP, Levi F (2004). Profile of women not attending in the Swiss Mammography Screening Pilot Programme. Breast.

[B25] Kreher NE, Hickner JM, Ruffin MT, Lin CS (1995). Effect of distance and travel time on rural women's compliance with screening mammography: an UPRNet study. Upper Peninsula Research Network. J Fam Pract.

[B26] Andreu Vaillo Y, Galdon Garrido MJ, Dura Ferrandis E, Carretero Gomez S, Tuells Hernandez J (2004). [Age, health beliefs, and attendance to a mammography screening program in the community of Valencia]. Rev Esp Salud Publica.

[B27] Nichols S, Koch E, Lallemand RC, Heald RJ, Izzard L, Machin D, Mullee MA (1986). Randomised trial of compliance with screening for colorectal cancer. BMJ.

